# Translation and Validation of the Japanese Version of the University of Washington Caregiver Benefit Scale and the Perception of Benefit-Finding by Caregivers of Children With Spina Bifida and Related Factors: Mixed Methods Research and Comparative Analysis

**DOI:** 10.2196/74069

**Published:** 2026-03-17

**Authors:** Xinmiao Cui, Tae Kawahara, Akemi Yamazaki

**Affiliations:** 1 Division of Health Sciences, Department of Pediatric and Family Nursing, Graduate School of Medicine, The University of Osaka, 1-7 Yamadaoka, Suita, Osaka, 565-0871, Japan, 81 6-6879-2537

**Keywords:** spina bifida, benefit finding, caregivers, parenting stress, social support

## Abstract

**Background:**

Spina bifida (SB) is a congenital condition that requires long-term multidisciplinary medical collaboration for treatment. Previous research has primarily focused on the negative impacts experienced by caregivers of children with SB. However, with the development of positive psychology, the concept of benefit-finding (BF) has been explored in the context of caregivers of children with various chronic illnesses. Nonetheless, in Japan, BF among caregivers of children with SB remains unexplored, and no appropriate measurement tool has been developed for this population.

**Objective:**

This study aimed to translate and validate the Japanese version of the University of Washington Caregiver Benefit Scale (UW-CBS) based on caregivers of children with SB and to examine the characteristics of BF in these caregivers. A comparative analysis with caregivers of able-bodied children was also conducted to elucidate the parenting stress and social support experienced by families rearing children with SB.

**Methods:**

This 2-part study was carried out from January 2024 to December 2024. In Study 1, the UW-CBS was translated, then face validity was examined through a pretest (n=6) using cognitive interviews. In the main survey, construct validity, known-groups validity, and retest reliability were evaluated (n=60). In Study 2, the characteristics of BF of caregivers of children with SB were analyzed using data from the main survey. Parents of able-bodied children (n=66) completed the same questionnaire. Parenting stress, BF, and social support scores were then compared between caregivers of children with SB and the parents of able-bodied children.

**Results:**

In Study 1, the reliability and validity of the UW-CBS were examined. Internal consistency was high (Cronbach *α*=0.92), while test-retest reliability had an intraclass correlation coefficient of 0.62 (*P*=.051). In Study 2, caregivers who had a partner (*P*=.009) and those who were rearing both a child with SB and a sibling reported higher levels of BF (*P*=.02). Compared with families rearing able-bodied children, no significant differences emerged in BF or parenting stress, but the level of social support was significantly higher in families of children with SB (*P*=.005).

**Conclusions:**

This study demonstrated the reliability and validity of the Japanese version of the UW-CBS in families rearing children with SB. For caregivers of children with SB, assistance from other family members or shared childcare responsibilities may facilitate positive adjustment. Moreover, the higher level of social support received by caregivers of children with SB may mitigate their parenting stress and foster their perception of benefits.

## Introduction

Spina bifida (SB) is a congenital disorder caused by incomplete closure of the neural tube that is generally accompanied by a variety of disorders, such as hydrocephalus, cognitive difficulties, bladder and bowel incontinence, and ambulatory dysfunction [1]. The management of SB requires not only multidisciplinary collaboration but also individualized care from caregivers because its clinical manifestations vary widely, with severity primarily determined by the level of spinal cord lesions. Caregivers of children with SB often face a range of challenges, having to balance the demands of long-term medical care, such as urinary and fecal incontinence [2], while also focusing on their child’s education and future [3].

Previous studies have primarily focused on the negative impacts of rearing children with SB, such as parenting stress [4,5]. A meta-analysis showed that families of children with SB and other chronic illnesses face the highest parental stress levels, highlighting the need for interventions aimed at improving marital relationships, enhancing social support, and equipping parents with strategies to manage parenting stress [6]. Compared with families with typically developing children, families of children with SB report higher levels of parenting stress related to managing children’s behavior, reflecting the challenges they face in addressing complex caregiving demands [7]. Further research has shown differences in stress sources between parents of children with SB: Fathers experience greater stress when dealing with children’s maladaptive behaviors and insufficient social support, whereas mothers’ stress is more closely related to the medical characteristics and dysfunction of the children [8]. Age also emerges as a significant factor affecting maternal parenting stress because older mothers report higher stress levels [9,10]. Among the medical factors, Kanaheswari et al [11] identified clean intermittent catheterization as the only significant contributor to maternal parenting stress in families of children with SB. Moreover, ongoing issues such as limited mobility and bladder and bowel dysfunction in school-aged children also contribute to persistent stress among parents of children with SB [12].

In the process of coping with parenting stress among caregivers of children with SB, social support may play a buffering role. Studies have shown that higher satisfaction with social support is closely associated with greater positive emotional adjustment in mothers [13]. Greater support from the family is also strongly linked to better physical health in mothers [14]. Furthermore, greater levels of social support are strongly related to fewer behavioral problems in children [8,15,16]. Notably, Feldman et al [16] demonstrated that social support plays a crucial moderating role between parenting stress and child adaptation. The aforementioned studies demonstrate that social support plays a critical role in multiple aspects of rearing children with SB. Meanwhile, further research underscores that, in pediatric rehabilitation settings, the importance of social support for families of children with SB cannot be overlooked [17]. In addition to mitigating negative emotions, social support contributes to the positive adjustment of caregivers. A study focused on caregivers of children with developmental disorders suggested that social support facilitates cognitive adaptation, thereby promoting positive emotional changes in caregivers [18].

Although caregiving challenges are substantial in families of children with SB, the demands associated with caregiving roles may create opportunities for personal growth and positive adaptation. Benefit-finding (BF) refers to the recognition of positive life changes that emerge from the process of coping with difficult life events [19]. Research on BF often focuses on the patients themselves [20-22], but caregivers of patients also experience BF. An increasing number of studies have reported positive changes, such as BF in caregivers of children with chronic illnesses [23-25]. In a qualitative study involving nuclear family members of children with chronic food hypersensitivity, a hierarchical BF process model was constructed. The findings suggested that parental modeling and reinforcement facilitated the sharing of positive experiences among family members [25]. This process highlights how BF emerges within families including children with chronic illness as a dynamic mechanism, where shared positive experiences not only strengthen individuals but also contribute to the overall harmony of the family unit.

In the context of SB, a study focused on young adults with SB found that the most prominent aspects of BF they experienced were related to personal strengths, interpersonal relationships, and life philosophy [26]. In addition, Kritikos et al [27] developed a BF scale specifically for individuals with SB based on a sample of 20 adolescents and young adults with SB. Although caregivers of children with SB are likely to experience more challenges, their special caregiving roles may also foster opportunities for positive psychological adaptation. However, research on BF by parents of children with SB remains limited, and the experiences of these families in different cultural contexts, such as Japan, have yet to be fully explored. Whereas the study by Kojo and Fukumaru [28] found that parents of children with SB tend to report high levels of depression, there remains a notable gap in understanding other characteristics, such as BF, parenting stress, and social support, of families of children with SB in the Japanese context.

In Japan, the existing caregivers’ BF scale was tailored specifically to caregivers of children with developmental disabilities [29], leaving a gap in measurement tools that adequately capture the BF experiences of caregivers of children with SB. The University of Washington Caregiver Benefit Scale (UW-CBS) developed by Amtmann et al [30] was designed to measure BF by caregivers regardless of whether their children have a medical condition. The flexible design of the UW-CBS makes it suitable for assessing BF in a variety of caregiving contexts.

Overall, this study sought to measure BF to explore the positive changes experienced by caregivers of children with SB. However, because no suitable scale exists in the Japanese context, the first objective of this research was to translate the UW-CBS into Japanese and evaluate its reliability and validity within the Japanese context. As little research in the Japanese context has focused on parenting stress, BF, and social support among caregivers of children with SB, the second goal of this study was to examine the current status of parenting stress, social support, and BF of caregivers of children with SB and clarify the unique characteristics of families of children with SB by including parents of able-bodied children as a comparison group.

## Methods

This research consisted of 2 parts and was carried out from January 2024 to December 2024.

### Study 1

#### Aim

Study 1 aimed to translate the UW-CBS [30] into Japanese and evaluate its reliability and validity using an anonymous cross-sectional questionnaire survey of caregivers of children with SB. To ensure the cultural equivalence and face validity of the UW-CBS, a small-scale pretest incorporating cognitive interviews was conducted, after which, the UW-CBS was revised for application in the main survey. Based on the Consensus-Based Standards for the Selection of Health Measurement Instruments (COSMIN) risk of bias checklist [31], an adequate sample size for cognitive interviews is 4 to 6 participants, and more than 30 participants is considered adequate for a quantitative main study.

#### Preparation

The International Society for Pharmacoeconomics and Outcomes Research [32] Good Practice for the Translation and Cultural Adaptation Process was adopted as the methodological framework. With the consent of the original author, the target population was defined as caregivers of children with SB. The research team consisted of 2 native Japanese speakers fluent in English with extensive experience in nursing research, together with a bilingual researcher involved in the study.

#### Forward Translation and Reconciliation

With the consent of the original author, 2 native Japanese speakers (TK and AY) independently translated the UW-CBS scale into Japanese. Discrepancies were discussed within the research team, and consensus was reached on the overall translation, with particular attention paid to the Japanese expressions of “feel closer” and “accepting person” and the use of sentence connectors to ensure natural and conceptual clarity. This reconciliation process resulted in Version 1, which was subsequently back-translated into English by the third bilingual researcher (XC). The back translation demonstrated conceptual equivalence with the original version.

#### Pretest and Cognitive Debriefing

Adult caregivers of children with SB were recruited using snowball sampling. After recruitment, questionnaires were mailed to the participating caregivers in advance, and subsequently, cognitive interviews were conducted with 6 caregivers via a videoconferencing application (Zoom Video Communications) or telephone. During this process, the time required to complete Version 1 of the UW-CBS was recorded. Using a verbal probing approach, caregivers were asked to comment on response ease, item clarity, discomfort in answering, relevance, and alignment with their experiences [33].

To explore caregivers’ BF experiences further, an open-ended question was included in the questionnaire, followed by approximately 30-minute interviews based on the caregivers’ responses. All interviews were audio-recorded and transcribed verbatim.

During the cognitive interviews, the caregivers indicated that the referent of “other person” was ambiguous. To address this issue, clarification was sought from the original author to ensure that the intended meaning was preserved in the translated version. Based on the feedback obtained, the research team discussed and refined Version 1 of the UW-CBS, resulting in Version 2.

Responses to the open-ended questions were qualitatively coded and categorized by one researcher (XC), with subsequent review and agreement within the research team.

#### Back Translation, Review, and Proofreading

Following an additional review by the research team to ensure wording and grammatical accuracy, back translation was performed by Editage, an independent professional translation service. Subsequently, the back translation was submitted to the original author for verification and formal approval.

#### Main Survey and Retest

Using Version 2 of the UW-CBS, the main survey was subsequently conducted (n=60). Caregivers of children with SB aged younger than 16 years were recruited through two methods. Caregivers undergoing treatment for depression or managing other children’s intensive care conditions were excluded. The caregivers’ responses were collected through both paper-based and online (Research Electronic Data Capture [REDcap] platform) methods. Among the caregivers of children with SB recruited through snowball sampling, 8 agreed to participate in the retest. The retest was conducted 2 weeks to 3 weeks after completion of the initial questionnaire and yielded 7 valid responses.

#### Questionnaire: Pretest

##### UW-CBS

The UW-CBS [30] is a publicly available, psychometrically sound instrument designed to measure the positive impacts of caregiving on adults. The UW-CBS is an item response theory–based tool composed of 13 items that evaluate the perceived benefits of caregiving, such as appreciation of life, discovering new strengths, and personal growth. Responses are rated from 1 (“Not at all”) to 5 (“Very much”), and scores are converted into an item response theory–based T-score to reflect BF levels. The UW-CBS has demonstrated good validity and reliability, with high test-retest reliability (intraclass correlation coefficient [ICC] >0.92) and strong internal consistency (Cronbach *α*=0.89). Moreover, to facilitate its use in diverse populations, the UW-CBS has been translated into Spanish, German, French, and Italian [34], demonstrating its potential as an internationally applicable tool for measuring the positive aspects of caregiving.

##### Open-Ended Responses

To investigate whether caregivers of children with SB experience unique aspects of BF, the following open-ended question was included: “While rearing a child with spina bifida, have you noticed any other aspects of your life where you have experienced positive changes?”

##### Parenting Stress Index-Short Form

The Japanese version of the Parenting Stress Index-Short Form (PSI-SF) [35], originally developed by Abidin as the Parenting Stress Index (PSI) [36], was used to assess parenting stress. The PSI-SF consists of 19 items rated on a 5-point scale and measuring 2 subdomains: parent-related (10 items) and child characteristic-related (9 items) stress scores. Responses range from 1 (“Not at all”) to 5 (“Exactly so”), with higher scores indicating higher levels of parenting stress. The PSI-SF has also been specifically adapted for use with Japanese parents to ensure cultural relevance.

##### Japanese Short Form of the Multidimensional Scale of Perceived Social Support

The Japanese short form of the Multidimensional Scale of Perceived Social Support (JMSPSS) [37] is derived from the original scale developed by Zimet et al [38], which uses a 7-point Likert-type scale ranging from 1 (“Not at all”) to 7 (“Very much”), with higher scores indicating greater perceived social support. The JMSPSS has a 3-factor structure: family support, significant other support, and friend support. The overall Cronbach α coefficient for reliability is 0.91, indicating good internal consistency.

##### Tachikawa Resilience Scale

The Tachikawa Resilience Scale (TRS) [39] is a 10-item self-reported measure designed to assess the resilience of Japanese individuals. In this study, the TRS was used to assess the construct validity of the UW-CBS. The TRS uses a 7-point Likert-type scale, ranging from 1 (“Not at all applicable”) to 7 (“Very applicable”), with higher scores indicating greater resilience. The Cronbach α coefficient of the TRS is 0.90, indicating good internal consistency.

##### Demographic Characteristics

Child-related items included age, sex, bladder condition (International Consultation on Incontinence Questionnaire-Urinary Incontinence Short Form, urinary incontinence, clean intermittent catheterization use and independence), bowel condition (bowel management and method, bowel movement independence, fecal incontinence, bowel management caregiver), mobility status, and shunt utilization of the child with SB; total number of children; and presence of significant illnesses in other children.

Caregiver-related items included age, relationship to the child, educational background, marital status, current mental illness, and self-reported life satisfaction.

### Data Analysis

The qualitative data from the pretest were transcribed verbatim, and interview data on BF experiences were analyzed using qualitative descriptive induction [40], which involves coding and categorization.

For the main survey, quantitative data were analyzed using SPSS for Windows (version 29; IBM Corp), with a significance level of *P*<.05. Descriptive statistics were used to summarize the demographic characteristics and the results from the administered scales. In this study, differential item functioning (DIF) was examined across gender and age using ordinal logistic regression, with McFadden *R*^2^<0.02 as the criterion [31]. Reliability was evaluated by calculating the Cronbach α coefficient for internal consistency and using the 2-way random effects (2,1) ICC for test-retest reliability. Construct validity was examined by analyzing the correlation between the UW-CBS and TRS. Given the conceptual relatedness between BF and resilience and prior evidence reported in the original study (*r*=0.28) [30], we hypothesized that the UW-CBS would be weakly positively correlated with the TRS. Known-groups validity was also evaluated. Caregivers of children with more severe medical conditions were hypothesized to have higher BF based on prior evidence that posttraumatic growth is higher in more severe and life-threatening medical contexts [41]. Similarly, caregivers with a spouse were hypothesized to have higher BF based on evidence linking marital adjustment and spousal support with greater resilience [42] and psychological health [43] among caregivers of children with chronic developmental conditions.

### Study 2

#### Aim

Study 2 sought to gain a deeper understanding of caregivers rearing children with SB in the main survey by including a control group consisting of the parents of able-bodied children. In the comparison, caregivers of children with SB constituted the SB group, and parents of able-bodied children constituted the able-bodied group. An anonymous cross-sectional survey was conducted to assess BF, parenting stress, and social support, with a subsequent analysis to compare differences between the two caregiver groups.

#### Comparison Between the SB Group and Able-Bodied Group

The members of the SB group were the participants in the main survey of Study 1 (n=60). Parents of able-bodied children (n=67) were recruited using the following 2 approaches: (1) a family-supporting specified nonprofit corporation (NPO) and (2) a participant recruitment company. The exclusion criteria included caregivers who were receiving treatment for depression or caring for their own children with conditions requiring intensive care. Parents recruited from the NPO responded to the questionnaire through REDcap.

#### Questionnaire

The same questionnaire used for the main survey of Study 1 was used. Regarding the UW-CBS, the Cronbach α coefficient in the able-bodied group was 0.96.

#### Data Analysis

We used 1-way ANOVA, independent samples *t* tests, Mann-Whitney *U* tests, and *χ*^2^ tests to measure the characteristics of caregivers of children with SB and the differences between the SB and able-bodied groups. Cohen *d* (≥0.8 for a large effect) indicated the effect size.

### Ethical Considerations

This study was approved by the Osaka University Hospital Ethics Committee (approval number: 23426‐4). Prior to participation, all participants received an information sheet explaining the purpose and procedures of the study, and informed consent was obtained through the consent confirmation section of the questionnaire. Participation was voluntary, and participants could withdraw from the study at any time without penalty. The questionnaire survey was conducted anonymously, and no personally identifiable information was collected. Data were managed using identification numbers and stored securely in locked cabinets and password-protected computers accessible only to the research team. Participants received a ¥500 (US $3.17) gift card as appreciation for their participation.

## Results

### Study 1

#### Pretest

##### Participants

In the pretest, 9 caregivers of children with SB were recruited; however, 1 was lost to follow-up, and 2 were unable to participate in the interview, resulting in a final sample of 6 caregivers. The 6 caregivers took approximately 2 minutes to complete the UW-CBS.

##### Validity Assessment

During the face validity cognitive interview, although a few concerns were raised about items 1, 2, 3, 5, and 12, the majority of caregivers indicated that these items were clear and easy to understand. For example, regarding the item “Are you a better advocate for your child/children because of caregiving?,” the caregivers mentioned that they felt confident in doing well when their child was younger. However, now that their child has his/her own opinions, they were uncertain about whether they fully and accurately convey their child’s intentions. For item 10, which used the Japanese translation of the term “accepting person,” most caregivers found the translation understandable but remarked that its phrasing felt slightly awkward. Thus, item 10, “accepting person,” was revised to more natural Japanese phrasing without altering its meaning. Overall, the caregivers indicated that most items were clearly expressed and successfully conveyed the intended meaning.

##### BF Experiences

Interviews with open-ended questions were conducted to explore the BF experiences that emerged among caregivers in the process of rearing their children with SB. The results identified several key aspects of BF: psychological and emotional growth, improvement of knowledge and skills, expansion of social support networks, changes in perspective, and planning for the future.

###### Psychological and Emotional Growth

For instance, caregivers initially used blogs as a way to gather information about SB and connect with other caregivers in similar situations. Over time, their focus shifted from seeking support to providing it:

*By starting the blog, I felt that I was able to connect with other children who have the same illness, and that it might also be helpful for children who will be born in the future.*

[Caregiver C]

###### Improvement of Knowledge and Skills

Caregivers not only actively sought information about SB itself but also developed an understanding of how to use assistive devices commonly needed by children with SB, such as wheelchairs and lower-limb orthoses. In addition, caregivers’ curiosity expanded beyond SB, leading them to explore information about children with other chronic conditions:

*Information about leg braces—such as the different types, their names, how to use them, and why they are worn…*

[Caregiver E]

###### Expansion of Social Support Networks

Caregivers expanded their social support networks by connecting with other parents of children with SB or chronic illnesses through activities such as their children’s participation in sports or blogging. These interactions provided some level of support and practical insights, which helped them navigate the challenges of caregiving more effectively:

*We talk about things like different types of wheelchairs. Even when there isn’t a specific problem to consult about, we stay connected on a regular basis.*

[Caregiver C]

###### Changes in Perspective

Rearing a child with SB gradually led caregivers to adopt new perspectives in certain areas. For example, caregivers began to see things from the perspective of disabilities; they stated that they focus on celebrating small daily achievements rather than comparing their child with others, emphasizing a mindset of progress and acceptance:

*It made me realize that there are so many different conditions out there, and I began to pay more attention to that.*

[Caregiver A]

###### Planning for the Future

Due to the unique needs of children with SB, caregivers found it necessary to make more detailed plans for the future, particularly regarding schooling and other key aspects of their child’s development:

*We had submitted a request, and arrangements were made so that an elevator would be properly installed in time for the child’s school enrollment.*

[Caregiver A]

### Main Survey

**Figure 1.**
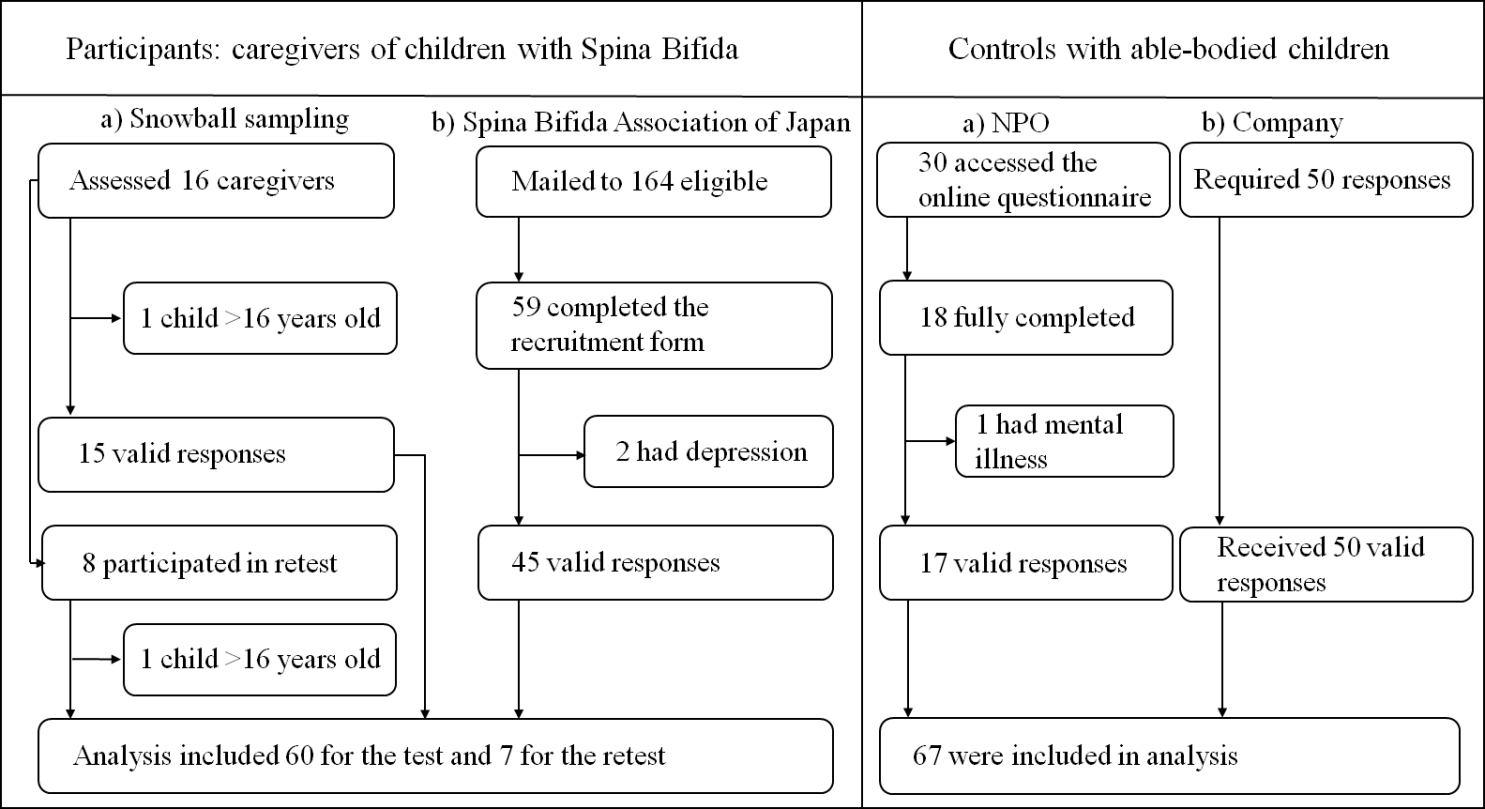
Recruitment processes in this study. NPO: specified nonprofit corporation.

Through snowball sampling, 16 caregivers of children with SB were recruited, 8 of whom participated in the retest of the UW-CBS, as shown in Figure 1. Two responses (1 main survey and 1 retest) were excluded because the child was older than 16 years.

The study introduction was sent by mail to 164 members of the Spina Bifida Association of Japan. Among these, 45 caregivers completed the questionnaire online (2 reported depression), while 2 responded via mail. The respondents’ demographic characteristics are summarized in Table 1. DIF was assessed across gender and age using ordinal logistic regression analysis, and all McFadden *R*^2^ values were <0.012.

**Table 1.** Demographic characteristics of caregivers (n=60) and demographic and clinical characteristics of children with spina bifida.

Characteristic	Results, n	UW-CBS^a^ score, mean
Caregivers’ age (years)	44.18^b^	N/A^c^
Relationship to child		
Mother	48	43.04
Father	12	43.83
Caregivers’ marital status		
Married	53	44.43
Divorced	6	34
Never married	1	33
Caregivers’ educational level		
High school or less	12	39.42
Vocational school or junior college	18	43.28
College or more	30	44.67
Caregivers’ life satisfaction		
Satisfied	13	46.77
Somewhat satisfied	35	43.31
Somewhat dissatisfied	9	38.56
Dissatisfied	3	40.33
Childs’ age (years)	10.72^b^	N/A
Childs’ sex		
Male	28	43.71
Female	32	42.75
Total number of children		
1	15	37.80
≥2	45	45
Ventriculoperitoneal shunt		
Yes	43	44.79
No	17	39.18
Clean intermittent catheterization (independence)		
Yes (independence)	38	42.45
No	15	45.73
Social urinary continence (≥4-hour dry period)		
Yes	27	43.56
No	33	42.91
Bowel management		
Yes	54	43.93
Person responsible for bowel management		
Child	14	42.57
Family and/or helper	40	44.40
No one	6	36.67
Mobility		
Community ambulator	36	43.19
Nonfunctional ambulator	3	49.33
Nonambulator	21	42.33

a UW-CBS: University of Washington Caregiver Benefit Scale.

b Mean.

c N/A: not applicable.

### Distribution

The analysis resulted in no ceiling nor floor effects across the 13 items (Table 2).

**Table 2.** Ceiling and floor effects of the overall University of Washington Caregiver Benefit Scale (UW-CBS) scores and for each of the 13 items as well as item-total correlations reported by caregivers of children with spina bifida (n=60).

Item content	UW-CBS score				Ceiling effect	Floor effect	Item-total correlation, *r*
	Mean (SD)		Median (range)				
Appreciate importance	3.60 (0.96)		4.00 (4.00)		4.56	2.64	0.59
Finding new strengths	3.42 (1.01)		3.00 (4.00)		4.43	2.41	0.43
Better advocate for children	3.10 (0.88)		3.00 (4.00)		3.98	2.22	0.50
Better person	2.92 (1.09)		3.00 (4.00)		4.01	1.83	0.67
Put life in perspective	3.22 (1.03)		3.00 (4.00)		4.25	2.19	0.66
More patient	3.60 (1.18)		4.00 (4.00)		4.78	2.42	0.67
Stronger person	3.68 (1.23)		4.00 (4.00)		4.91	2.45	0.72
Gained confidence	2.83 (1.22)		3.00 (4.00)		4.05	1.61	0.73
Add meaning to life	3.73 (1.13)		4.00 (4.00)		4.86	2.60	0.76
More accepting person	3.32 (1.16)		3.00 (4.00)		4.48	2.16	0.75
More caring	3.67 (1.12)		4.00 (4.00)		4.79	2.56	0.79
Closer to other adults	3.25 (0.93)		3.00 (4.00)		4.18	2.32	0.68
Closer to partner	2.88 (1.43)		3.00 (5.00)		4.31	1.45	0.58
Total scores on the UW-CBS	43.20 (10.31)		44.00 (45.00)		53.51	32.90	N/A^a^

a Not applicable.

### Reliability

The Cronbach α coefficient for the UW-CBS was 0.92. A total of 7 participants completed the retest, and the ICC yielded a result of 0.62 (*P*=.051).

### Validity

The correlation between the TRS and UW-CBS was *r*=0.30 (*P*=.02), aligning with the hypothesis. Known-groups validity was examined by comparing UW-CBS total scores across groups defined by children’s medical severity and caregivers’ marital status. In both comparisons, the groups hypothesized to have greater BF showed higher UW-CBS total scores (Table 3).

**Table 3.** Independent samples *t* test and Mann-Whitney *U* test for known-groups validity.

Characteristic	Sample size, n	UW-CBS^a^ score, mean	Statistical test result	*P* value
Ventriculoperitoneal shunt			230^b^	.03
Yes	43	44.79		
No	17	39.18		
Caregivers’ relationship status			2.68 (58)^c^	.009
With a partner	53	44.43		
Without a partner	7	33.86		

a UW-CBS: University of Washington Caregiver Benefit Scale.

b Mann-Whitney *U* test.

c *t* test (*df*).

### Study 2

#### Characteristics of BF in Families of Children With SB

The UW-CBS scores for each characteristic are presented in Table 1. Two results with significant differences were identified: Single mothers reported lower UW-CBS scores (*t*_58_=2.68, *P*=.009; *d*=0.95), and caregivers rearing only children with SB reported lower UW-CBS scores (*t*_58_=2.40, *P*=.02; *d*=0.75). The results are shown in Table 4.

**Table 4.** Characteristics of caregivers (n=60) of children with spina bifida with regard to University of Washington Caregiver Benefit Scale (UW-CBS) scores.

Characteristic	Sample size, n	UW-CBS score, mean (SD)	*t* value (*df*)	*P* value	Cohen *d*
Total number of children			2.40 (58)	.02	0.75
1	15	37.80 (9.18)			
≥2	45	45.00 (10.12)
Caregivers’ relationship status			2.68 (58)	.009	0.95
With a partner	53	44.43 (9.45)			
Without a partner	7	33.86 (12.48)

#### Correlations Between BF, Parenting Stress, and Social Support Among Families of Children With SB

The JMSPSS had a significant positive correlation with the UW-CBS (*r*=0.30, *P*=.02) and a significant negative correlation with the PSI-SF (*r*=–0.47, *P*<.001). Although the correlation between the UW-CBS and the PSI-SF was not statistically significant, a negative association was observed (*r*=–0.22, *P*=.90).

#### Comparison Between the SB and Able-Bodied Groups

##### Comparison of Demographic Characteristics

For the able-bodied group, a total of 18 participants completed the questionnaire through recruitment by the NPO. One answer was excluded because the respondent reported a mental illness but declined to disclose the specific diagnosis. In addition, 50 parents of able-bodied children were recruited through the company. To ensure comparability, the characteristics of the SB and able-bodied groups were compared, but no significant differences were found.

##### Comparison of Scales

The results of the comparison of each scale between the groups are shown in Table 5. The analysis comparing the 2 groups revealed no significant difference in UW-CBS nor PSI-SF scores, but the SB group had higher JMSPSS scores (*U*=1428.50, *P*=.005).

**Table 5.** Results regarding the distribution of and the Mann-Whitney *U* or independent samples *t* tests of the University of Washington Caregiver Benefit Scale (UW-CBS), Parenting Stress Index-Short Form (PSI-SF), and Japanese short form of the Multidimensional Scale of Perceived Social Support (JMSPSS) scores between the spina bifida (SB; n=60) and able-bodied (n=67) groups.

Scale	SB group		Able-bodied group		Mean difference	
	Mean	Median	Mean	Median	Statistical test result	*P* value
UW-CBS	43.20	44.00	41.25	42.00	1885.50^a^	.55
PSI-SF total scores	43.68	42.50	43.94	45.00	0.12 (125)^b^	.90
Child characteristic-related stress scores^c^	21.58	22.00	20.46	21.00	1726.50^a^	.17
Parent-related stress scores^c^	22.10	22.00	23.48	23.00	1.11 (125)^b^	.27
JMSPSS	65.23	66.00	57.82	60.00	1428.50^a^	.005

a Mann-Whitney *U* test.

b Independent *t* test (*df*).

c Subscale of the PSI-SF.

## Discussion

### Study 1

The aim of Study 1 was to translate the UW-CBS into Japanese and validate its reliability and validity through a pretest and main survey. Because the UW-CBS was not developed in an Asian context, cognitive interviews were conducted as a critical step to address potential cultural differences in the process of scale adaptation. Overall, nearly all caregivers were able to understand the meaning of all items and found most of them to be clearly expressed. However, caregivers also experienced some hesitation and confusion while responding to certain items. Similar feedback was also provided when the UW-CBS was translated into European languages [34], suggesting that, although the scale is broadly applicable, some conceptual nuances require careful adaptation.

Regarding reliability, internal consistency was high (Cronbach *α*=0.92), but test-retest reliability yielded an ICC of 0.62 (*P*=.051), which did not reach the level of statistical significance. This marginal result likely reflects a lack of statistical power due to the small sample size (n=7) rather than the inherent instability of the scale. However, given that the original version demonstrated robust stability and our qualitative findings confirmed the items’ conceptual clarity, these results provide a preliminary indication of the scale’s reliability.

In adapting the UW-CBS to Europe, the mean score (44.50) was lower than that in the United States (47.90) [30]. In Japan, this study yielded an even lower mean score (43.20). In addition to the issues caregivers encountered while responding, this phenomenon may also be attributed to the cultural differences between Asian and Western contexts. Compared with those in the United States, Japanese adults report higher levels of pessimism and are less positive [44]. In addition, adults in the United States perceive self-esteem as both desirable and consequential, whereas Japanese people view self-esteem as desirable but not consequential [45]. Japanese respondents tend to use rating scales more moderately [46], which may also lead to lower observed mean scores. These differences in cultural perspectives may partially explain why the UW-CBS scores observed in this study were lower in the Japanese context, as a more pessimistic outlook and less emphasis on self-esteem may reduce the likelihood of identifying benefits in caregiving situations. In addition, interviews with caregivers about their BF experiences while rearing children with SB revealed several recurring themes, including psychological and emotional growth, improved knowledge and skills, expanded social support networks, changes in perspective, and planning for the future. The results of the semistructured interviews on BF indicated that experiences related to BF exist within caregivers of children with SB, and the themes appear to align with the content of most items on the UW-CBS, suggesting a potential connection between the scale’s construction and the lived experiences of the target population.

In the main survey, no ceiling nor floor effects were observed for the 13 items on the UW-CBS, and the total score followed a normal distribution. The absence of meaningful DIF across gender and age, indicated by McFadden *R*^2^ values <0.012, suggests that the UW-CBS demonstrates measurement stability. In addition, the overall Cronbach α coefficient for the Japanese-translated UW-CBS was 0.92. Whereas the original study reported that this correlation was lower than anticipated, the result obtained in this study was similar to the original result. A retest with 7 caregivers was conducted, and the test-retest reliability was 0.62, lower than that in the original study (0.92). Although the result in this study was not significant, likely because of the small sample size, the ICC of 0.62 still suggests that the UW-CBS demonstrates a certain degree of reliability. Construct validity was examined by exploring the correlation between the resilience scale and the UW-CBS. In this study, the correlation was *r*=0.30 (*P*=.02), which is comparable to the original study’s correlation of *r*=0.28 (*P*<.01). The known-groups validity comparisons identified differences in BF between caregivers of children with SB who differed in medical condition, as indicated by ventriculoperitoneal shunt use, as well as between caregivers with and without a partner, in the expected directions. These findings suggest that the UW-CBS is capable of capturing meaningful variation in BF across caregiver subgroups.

### Study 2

The aim of Study 2 was to explore the characteristics of caregivers of children with SB. Because little research has explored BF among caregivers of children with SB, we first examined the characteristics of their scores on the UW-CBS. We found that parents rearing only children with SB scored lower on the UW-CBS than did those who were also rearing other children without serious conditions. This result suggests that a greater number of children may offer caregivers increased emotional support or family resources, which may foster more positive emotional experiences. Previous research concerning parents of children with autism spectrum disorder has indicated that parents and siblings themselves believe that they mature more quickly as a result of taking on a caregiver role [47]. Furthermore, in a study of resilience among families of children with autism, interviewers also noted that siblings tend to look after the child with autism, thereby bringing family members closer together [48]. These findings may also be applicable to families rearing children with SB, where the presence of additional children may contribute to the development of a stronger support network among family members, thereby influencing caregivers’ perceptions of BF.

In this study, caregivers with partners had higher UW-CBS scores. This result aligns with previous research emphasizing the importance of marriage, as marital satisfaction contributes to improved psychological adjustment [6] and positive child adjustment outcomes [49]. Another study highlighted that a partner’s companionship is an indispensable source for mothers of children with SB, which cannot be easily substituted by other forms of social support [50]. By contrast, single mothers of children with SB reported significantly higher parenting stress [7,11], suggesting that the absence of a partner may exacerbate parenting challenges. Further research should place greater emphasis on providing support for single-parent families of children with SB.

The comparison between caregivers of children with SB and parents of able-bodied children resulted in a significant difference in social support scores, but no significant differences were observed in parenting stress nor BF. The significant difference in social support may stem from the extensive medical needs of children with SB, which often require ongoing help from health care professionals and social workers. In addition, caring for a child with SB can lead caregivers to connect with others in similar situations, further expanding their support networks. These assumptions are partially supported by the pretest findings, in which the category *Expansion of social support networks* emerged. Caregivers mentioned having access to help from specialists in areas like rehabilitation and welfare during difficult times, as well as forming friendships with other caregivers of children with SB.

In prior studies, caregivers of children with SB reporting higher levels of social support also tended to report lower parenting stress [8,16] and distress [13]. Despite the fact that parents of children with SB have generally reported higher levels of parenting stress in most studies [6-12], greater social support may account for the finding in this study that caregivers of children with SB experience levels of parenting stress comparable to those of parents rearing able-bodied children.

Although the original UW-CBS paper reported higher BF scores in community samples compared with those in medical condition samples [30], in this study, no significant difference in UW-CBS scores was found between caregivers of children with SB and the control group. This result may be attributed to the relatively high levels of social support received by the caregivers of children with SB in this study. A study focusing on caregivers of children with developmental disabilities explored a BF-related model, identifying a positive relationship between social support and BF while suggesting that social support nourishes framework reconstruction and potentially facilitates the BF process [18]. This finding raises the possibility that caregivers of children with SB, as fellow caregivers of children with chronic conditions, might experience a greater degree of BF in the context of a well-developed social support network.

### Limitations

This study has some limitations. First, the recruitment of caregivers was inherently challenging because of the medical complexity and care demands associated with SB, and recruitment was further constrained by the COVID-19 pandemic. As a result, the sample sizes for the main survey and retest were relatively small, which limited the statistical power. In particular, test-retest reliability did not achieve statistical significance, suggesting that the results regarding the scale’s stability should be interpreted as preliminary. Second, the validation of the scale was restricted to caregivers of children with SB as the target group. In future research, the application of the UW-CBS is anticipated to be examined in larger sample sizes and among a broader range of caregivers of children with special needs to evaluate the stability and measurement properties of the UW-CBS. Second, because this study had a cross-sectional design and measured BF at a specific point in time, longitudinal research should be considered to gain a deeper understanding of the positive psychological adaptation process of parents of children with SB within the Japanese context. Third, the recruitment strategy, which relied on associations and snowball sampling, may have led to an overrepresentation of caregivers with higher levels of social support. This may reduce the representativeness of the study sample. Future studies should adopt broader and more systematic recruitment strategies to enhance representativeness and reduce potential selection bias.

### Conclusions

This study, based on the context of Japanese caregivers of children with SB, translated and validated the UW-CBS, demonstrating its usability. In addition, BF was observed in families of children with SB, with more pronounced levels in families simultaneously rearing typically developing children and in households with a spouse or partner. Families of children with SB also reported receiving greater levels of social support than parents of able-bodied children.

## Abbreviations

BF: benefit-finding
COSMIN: Consensus-Based Standards for the Selection of Health Measurement Instruments
DIF: differential item functioning
ICC: intraclass correlation coefficient
JMSPSS: Japanese short form of the Multidimensional Scale of Perceived Social Support
NPO: specified nonprofit corporation
PSI-SF: Parenting Stress Index-Short Form
REDcap: Research Electronic Data Capture
SB: spina bifida
TRS: Tachikawa Resilience Scale
UW-CBS: University of Washington Caregiver Benefit Scale
